# Mechanical Unloading of Engineered Human Meniscus Models Under Simulated Microgravity: A Transcriptomic Study

**DOI:** 10.1038/s41597-022-01837-x

**Published:** 2022-11-30

**Authors:** Zhiyao Ma, David Xinzheyang Li, Ryan K. W. Chee, Melanie Kunze, Aillette Mulet-Sierra, Mark Sommerfeldt, Lindsey Westover, Daniel Graf, Adetola B. Adesida

**Affiliations:** 1 Department of Surgery, Division of Orthopaedic Surgery, Faculty of Medicine & Dentistry, University of Alberta, Edmonton, AB T6G 2E1 Canada; 2 Department of Civil & Environmental Engineering, University of Alberta, Edmonton, AB Canada; 3 Department of Mechanical Engineering, University of Alberta, Edmonton, AB Canada; 4 School of Dentistry, University of Alberta, Edmonton, AB Canada

**Keywords:** Mechanisms of disease, Data acquisition

## Abstract

Osteoarthritis (OA) primarily affects mechanical load-bearing joints, with the knee being the most common. The prevalence, burden and severity of knee osteoarthritis (KOA) are disproportionately higher in females, but hormonal differences alone do not explain the disproportionate incidence of KOA in females. Mechanical unloading by spaceflight microgravity has been implicated in OA development in cartilaginous tissues. However, the mechanisms and sex-dependent differences in OA-like development are not well explored. In this study, engineered meniscus constructs were generated from healthy human meniscus fibrochondrocytes (MFC) seeded onto type I collagen scaffolds and cultured under normal gravity and simulated microgravity conditions. We report the whole-genome sequences of constructs from 4 female and 4 male donors, along with the evaluation of their phenotypic characteristics. The collected data could be used as valuable resources to further explore the mechanism of KOA development in response to mechanical unloading, and to investigate the molecular basis of the observed sex differences in KOA.

## Abstract (summary)

**Table:** 

Measurement(s)	Transcriptome
Technology Type(s)	mRNA Sequencing
Factor Type(s)	Mechanical stimulation
Sample Characteristic - Organism	Homo sapiens

## Background & Summary

Osteoarthritis (OA) is a degenerative joint disease that primarily affects mechanical load-bearing joints, with the knee being the most common^1,2^. Much of the focus of OA is on knee osteoarthritis (KOA) due to its 83% prevalence among cases of OA^3,4^. Most demographic groups are affected by OA, but the prevalence and burden are disproportionately higher in females^2,5–10^. While sex hormones regulate joints’ cartilage and bone development and homeostasis in a sex-dependent manner, hormonal differences alone do not fully account for the higher incidence of OA in females^8^. Kinney *et al*. reported that sex-specific variations in the response of human articular chondrocytes to estrogen are due to differences in receptor number and the mechanisms of estrogen action^11^.

The molecular basis for sex differences in the burden of KOA is not well understood, and questions remain for the cellular and molecular events underlying the pathogenesis and progression of KOA. However, some cellular and molecular characteristics of KOA resemble chondrocyte hypertrophy before endochondral ossification during skeletogenesis^12^. This includes chondrocyte proliferation, chondrocyte hypertrophy along with upregulation of hypertrophy markers *COL10A1*^13^ and *MMP13*^14^, remodelling of the cartilage matrix by proteases, vascularization, and focal calcification of cartilage with hydroxyapatite crystals.

A plethora of *in vitro* and *in vivo* studies show joint cartilage atrophy after long-term mechanical unloading (i.e., joint immobilization)^15–24^. For example, in a case study involving ten healthy young individuals (4 males and 6 females), with no history of KOA requiring 6 to 8 weeks of non-weight bearing for injuries affecting the distal lower extremity, the axial mechanical unloading of the joint resulted in increased magnetic resonance imaging (MRI) parameters, *T1rho* and *T2* relaxation times, of the knee articular cartilage that resembled signs of KOA. After four weeks of returning to axial mechanical loading, the *T1rho* and *T2* relaxation times were restored to baseline values of normal healthy articular cartilage^21^. However, it is unknown if the magnitude or rate of cartilage atrophy was disproportionate between the male and female participants after mechanical unloading. And neither were knee menisci investigated despite their functional importance for load distribution across the knee joint.

The effects of mechanical unloading on cartilage have been studied via simulated microgravity (SMG)^22,23,25–27^. Microgravity, both real and simulated, as well as reduced weight-bearing, have been shown to induce detrimental effects on cartilage, and in some cases, promote an OA-like phenotype^16,19,20,23,24,28,29^. This makes SMG a relevant model to study OA-related changes in chondrocytes from mechanical unloading conditions^23,25^. SMG can be produced by rotating wall vessel (RWV) bioreactors developed by the National Aeronautics Space Administration (NASA). RWV rotates at a constant speed to maintain tissues in a suspended free-fall, resulting in a randomized gravitational vector^24,30^. Our group has demonstrated that four weeks of SMG using a RWV bioreactor could enhance KOA-like gene modulations in bioengineered cartilage^26^. Specifically, *COL10A1* and *MMP13* as markers of chondrocyte hypertrophy were significantly increased by SMG^26^. However, the potential of mechanical unloading and molecular profiling techniques to explore the molecular basis of the disproportionate incidence of KOA in females is yet to be explored. Menisci from KOA joints have been reported to exhibit similar molecular characteristics found in osteoarthritic articular cartilage^31,32^. Osteoarthritic meniscus fibrochondrocytes (MFC) produced more calcium deposits than normal MFC^32,33^. Moreover, osteoarthritic MFC expressed aggrecan (*ACAN*) at a significantly higher level than normal MFC^33^. More recently, type X collagen and MMP-13 were shown to be highly expressed in osteoarthritic meniscus relative to the normal meniscus^31^. As both proteins are markers of chondrocyte hypertrophy, these findings suggested that MFC undergo hypertrophic differentiation like osteoarthritic articular chondrocytes and that the knee menisci maybe actively involved in the pathogenesis of OA. The potential impact of OA on meniscus tissue was also explored in a mice model that was exposed to either microgravity on the International Space Station or hind limb unloading^16^. Prolonged unloading in both treatments resulted in cartilage degradation, meniscal volume decline, and elevated catabolic enzymes such as MMPs^16^.

In this study, meniscus models were generated from healthy human meniscus MFC seeded onto a type I collagen scaffold and cultured under SMG condition in RWV bioreactor with static normal gravity as controls. Full transcriptome RNA-sequencing of 4 female and 4 male donors were conducted, along with the analysis of phenotypic characteristics. Data from this study could be used to further explore the mechanism of the early onset of KOA and to investigate the molecular basis of observed sex differences in KOA.

## Methods

**Fig. 1:**
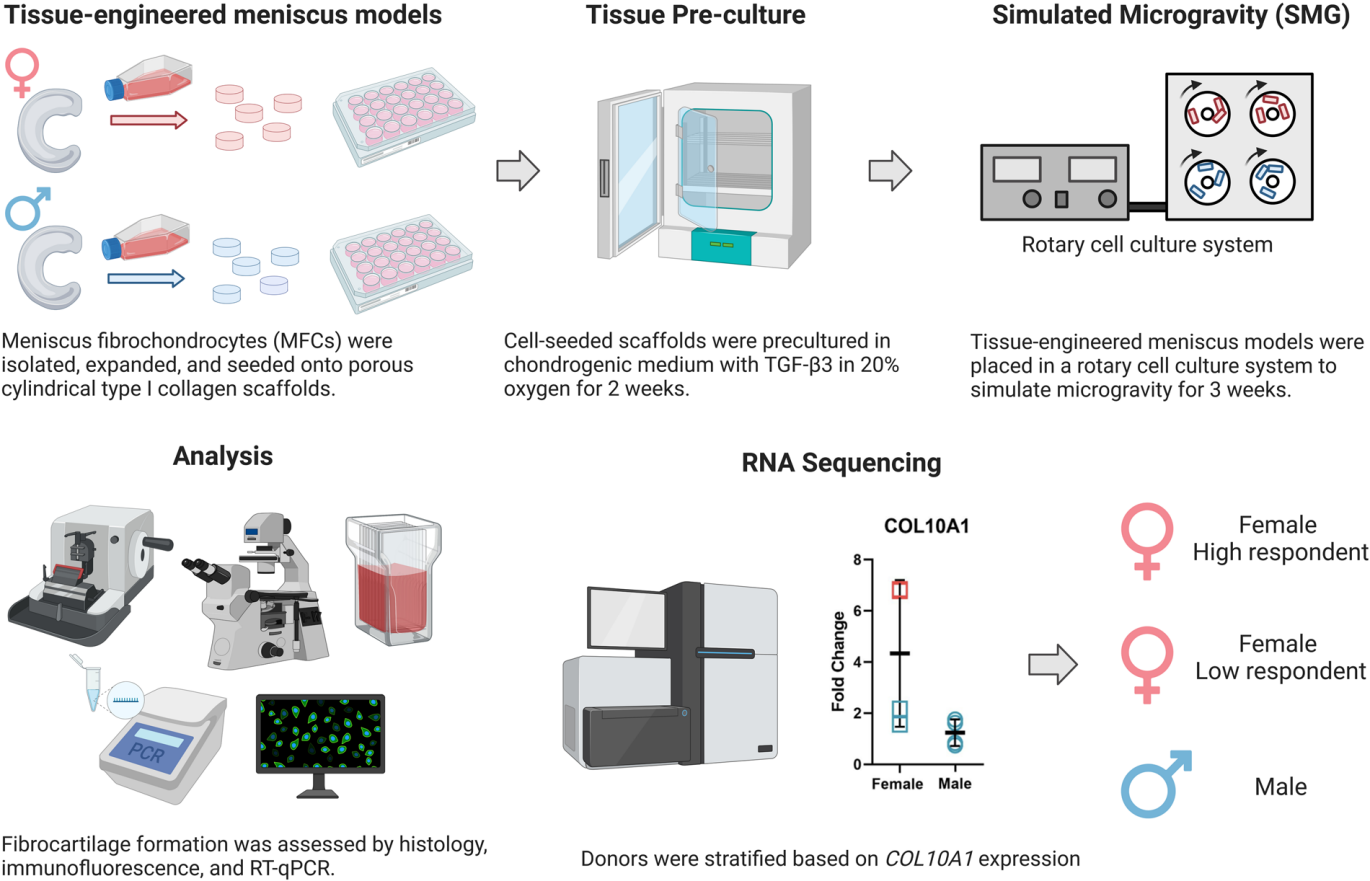
Experiment overview. Human meniscus fibrochondrocytes (MFC) were isolated from 4 males and 4 females. Complete treatment was repeated for each donor. Created with BioRender.com.

The experiment is outlined in Fig. 1. Most culture methods and assays were performed identically to those described in previous work^34–36^.

### Ethics statement

Human non-osteoarthritic inner meniscus samples were obtained from patients undergoing arthroscopic partial meniscectomies because of traumatic meniscal tears at the Grey Nuns Community Hospital in Edmonton. Experimental methods and tissue collection were with the approval of and in accordance with the University of Alberta’s Health Research Ethics Board-Biomedical Panel (Study ID: Pro00018778). The ethics board waived the need for written informed consent of patients, as specimens used in the study were intended for discard in the normal course of the surgical procedure. Extensive precautions were taken to preserve the privacy of the participants donating specimens such that only patient sex, age, weight, height, and underlying health conditions were provided.

### Cell isolation

Meniscus fibrochondrocytes (MFC) were isolated from inner meniscus specimens by digestion with type II collagenase (0.15% w/v of 300 units/mg; Worthington). For this, the inner meniscus specimens were first cut into smaller pieces. The type II collagenase solution in high glucose Dulbecco’s modified Eagle’s medium (HG-DMEM) supplemented with 5% (v/v) fetal bovine serum (FBS) was added to pieces at a ratio of 10 mL per 1 g of the meniscus and left to incubate at 37 °C for 22 hours in an orbital shaker set at 250 rpm. After digestion, the cell suspension was filtered through a 100-μm nylon mesh filter (Corning Falcon, NY, USA), and MFC were isolated by centrifuge. The isolated MFC were plated for 48 hours to recovery before cell expansion.

### Cell expansion

After 48 hours, the MFC were detached by 0.05% w/v trypsin-EDTA in Hank’s buffered saline solution (Invitrogen, CA, USA) and plated in a tissue culture flask (Sarstedt, Germany) at 10^4^ cells/cm^2^ in HG-DMEM supplemented with 10% v/v heat-inactivated fetal bovine serum (FBS), 100 mM 4-(2-hydroxyethyl)-1-piperazineethanesulfonic acid (HEPES), 1 mM sodium pyruvate (all from Sigma-Aldrich Co., MO, USA), and 100 U/mL penicillin, 100 µg/mL streptomycin and 0.29 mg/mL glutamine (PSG; Life Technologies, ON, Canada), and 5 ng/mL of FGF-2 (Neuromics, MH, USA, catalog #: PR80001) and 1 ng/mL of TGF-β1 (ProSpec, catalog #: CYT-716) for 1 week. Cell population doubling (PD) was calculated as the log_2_(*N/N*_*O*_), where *N*_*O*_ and *N* are the number of MFC respectively at the beginning and the end of the cell amplification period.

### 3D Cell culture in porous type I collagen scaffold

The expanded MFC were resuspended in a defined serum-free chondrogenic medium HG-DMEM supplemented with HEPES, PSG, ITS + 1 premix, 125 μg/mL of human serum albumin, 100 nM of dexamethasone, 365 μg/mL ascorbic acid 2-phosphate, 40 μ/mL of L-proline, and 10 ng/mL of TGF-β3, followed by seeding onto type I collagen scaffolds (diameter: 6 mm or 10 mm; height: 3.5 mm; pore size: 115 ± 20 μm, Integra LifeSciences, NJ, USA) at the density of 5 × 10^6^ cells/cm^3^. The cell-seeded scaffolds were cultured in a 24-well plate (12-well plate for 10 mm scaffolds) with serum-free chondrogenic medium for 2 weeks for initial extracellular matrix formation. Medium change was performed once per week.

### Mechanical stimulation

After 2 weeks of preculture, engineered meniscus tissues of each donor were randomly assigned to two mechanical stimulation groups: static control under normal gravity and simulated microgravity (SMG). Each experimental group had 8 technical replicates of engineered tissue constructs. For the static control group, engineered constructs were cultured in a tissue culture tube (Sarstedt, Germany) with 55 mL serum-free chondrogenic medium for 3 weeks. For the SMG group, the same number of constructs were cultured in the slow turning lateral vessels (STLV; Synthecon, Inc., TX, USA) on a rotary cell culture system (RCCS-4; Synthecon, Inc.) with 55 mL of serum-free chondrogenic medium for 3 weeks. The rotation speed of the STLV was adjusted during the 3-week treatment to account for the increasing weight of the tissues and to maintain a stable free-falling position (30 rpm from day 1 to day 2, 34 rpm from day 3 to day 7, 37 rpm day from 8 to day 13, 40 rpm day from 14 to day 21). Medium change was performed for both groups once per week.

### Histology and immunofluorescence

After 3 weeks of mechanical stimulation, constructs intended for histology and immunofluorescence were fixed in 1 mL of 10% v/v buffered formalin (Fisher Scientific, MA, USA) overnight at 4 °C, dehydrated, and embedded in paraffin wax. The embedded tissues were sectioned at 5 μm thickness and stained for Safranin O, Fast Green, Haematoxylin, collagen type I, collagen type II, and collagen type X.

For immunofluorescence staining, the sections were first deparaffinized and rehydrated. Protease XXV (Thermo Scientific) and hyaluronidase (Sigma-Aldrich) were then applied for 30 min each to improve antigen accessibility. Blocking was performed with 5% w/v bovine serum albumin in PBS for 30 min before staining for the primary antibody. Collagen type I, type II, and type X were stained with a 1:200 dilution of rabbit anti-collagen I antibody (CL50111AP-1; Cedarlane Labs, ON, Canada), a 1:200 dilution of mouse anti-collagen II antibody (II-II6B3, Developmental Studies Hybridoma Bank, IA, USA), and a 1:100 dilution of rabbit anti-collagen X antibody (ab58632; Abcam, UK), respectively. The sections were incubated with primary antibodies overnight at 4 °C. Secondary antibody labelling was applied on the second day. A 1:200 goat anti-rabbit IgG (H&L Alexa Fluor 594; Abcam) was used for collagen I and X. A 1:200 goat anti-mouse IgG (H&L Alexa Fluor 488; Abcam) was used for collagen II. Cell nuclei were stained with DAPI (4′, 6-diamidino-2-phenylindole; Cedarlane) after secondary antibody staining. The slides were finally mounted with a 1:1 ratio of glycerol and PBS. The Eclipse Ti-S microscope (Nikon Canada; ON, Canada) was used for immunofluorescence images.

### RNA extraction, RT-qPCR, and RNA Sequencing

Constructs intended for transcriptome analysis were preserved in Trizol (Life Technologies) immediately upon harvesting and stored at −80 °C until RNA extraction. For female and male donors # 1, 2, and 3, RNA was extracted and purified from ground samples using PuroSPIN Total DNA Purification KIT (Luna Nanotech, Canada) following the manufacturer’s protocol. For female and male donors # 4, RNA was extracted and purified from ground samples using RNeasy Minikits (Qiagen, ON, Canada) following the manufacturer’s protocol. The quality of purified RNA was assessed by Nanodrop One (Thermo Scientific, USA), and fixed mass of RNA were sent for RNA sequencing at the University of British Columbia Biomedical Research Centre (UBC-BRC). A standard quality control assessment was conducted on the RNA samples prior to sequencing. All RNA samples showed reasonable quality and no noticeable differences were observed in quality between the two isolation methods. Next-generation sequencing was performed on the Illumina NextSeq. 500 with paired-end 42 bp × 42 bp reads and FastQ files were obtained from sequenced donors for further bioinformatics analysis.

Extracted RNA was reverse transcribed into cDNA with GoScript reverse transcriptase (Fisher Scientific) and amplified by quantitative real-time polymerase chain reaction (RT-qPCR) for chosen genes with specific primers (Table 1). The expression level of selected genes was normalized to chosen housekeeping genes (Table 1; *B-actin*, *B2M*, and *YWHAZ*) based on the coefficient of variation (CV) and M-value as measures of reference gene stability^37^, and the data was presented using the 2^−∆∆CT^ method^38,39^. An unpaired *t*-test was performed between treatment groups.

**Table 1:** Real-time qPCR Primer Sequences.

Gene	Forward	Reverse	GenBank Accession
ACAN	AGGGCGAGTGGAATGATGTT	GGTGGCTGTGCCCTTTTTAC	NM_001135.3
Β-actin	AAGCCACCCCACTTCTCTCTAA	AATGCTATCACCTCCCCTGTGT	NM_001101.4
B2M	TGCTGTCTCCATGTTTGATGTATCT	TCTCTGCTCCCCACCTCTAAGT	NM_004048.3
COL1A2	GCTACCCAACTTGCCTTCATG	GCAGTGGTAGGTGATGTTCTGAGA	NM_00008 9.3
COL2A1	CTGCAAAATAAAATCTCGGTGTTCT	GGGCATTTGACTCACACCAGT	NM_001844.5
COL10A1	GAAGTTATAATTTACACTGAGGGTTTCAAA	GAGGCACAGCTTAAAAGTTTTAAACA	NM_000493.3
SOX9	CTTTGGTTTGTGTTCGTGTTTTG	AGAGAAAGAAAAAGGGAAAGGTAAGTTT	NM_000346.3
YWHAZ	TCTGTCTTGTCACCAACCATTCTT	TCATGCGGCCTTTTTCCA	NM_003406.3

### Bioinformatics and donor stratification

Next-generation sequencing data were analyzed with Partek® Flow® software (Version 10.0.21.0302, Copyright © 2021, Partek Inc., St. Louis, MO, USA). A quality score threshold of 20 was set to trim the raw input reads from the 3′ end. Trimmed data were then aligned to the reference human genome hg38 using the STAR 2.7.3a aligner and followed by the quantification to a transcript model (hg38-RefSeq Transcripts 94 - 2020-05-01) using the Partek E/M algorithm. A noise reduction filter was applied to exclude genes whose maximum read count was below 50. Quantified and filtered reads were then normalized using the Add: 1.0, TMM, and Log 2.0 methods in sequential order. Based on the fold change of *COL10A1* expression level (SMG to static), sequenced female donors were stratified into a high-response group and a low-response group, while male donors remained in one group for further analysis. Statistical analysis was performed using analysis of variance (ANOVA) for sex and treatment. Differentially expressed genes (DEGs) for each comparison were determined by *p*-value and fold change (FC). Principal component analysis (PCA) and the visualization of DEGs using Venn diagrams were all conducted in Partek® Flow® software.

## Data Records

All data generated during this study are deposited in the National Centre for Biotechnology Information (NCBI) Gene Expression Omnibus (GEO) with accession number GSE192983. The deposited data includes the raw FastQ files and.txt files containing normalized counts generated as described in the method section. The raw RNA Sequencing dataset can be found in the online repository^40^. The histology and immunofluorescence figures of each treatment group for all donors can be accessed at Figshare^41^.

## Technical Validation

### Phenotypes of engineered meniscus constructs under different culture conditions

**Fig. 2:**
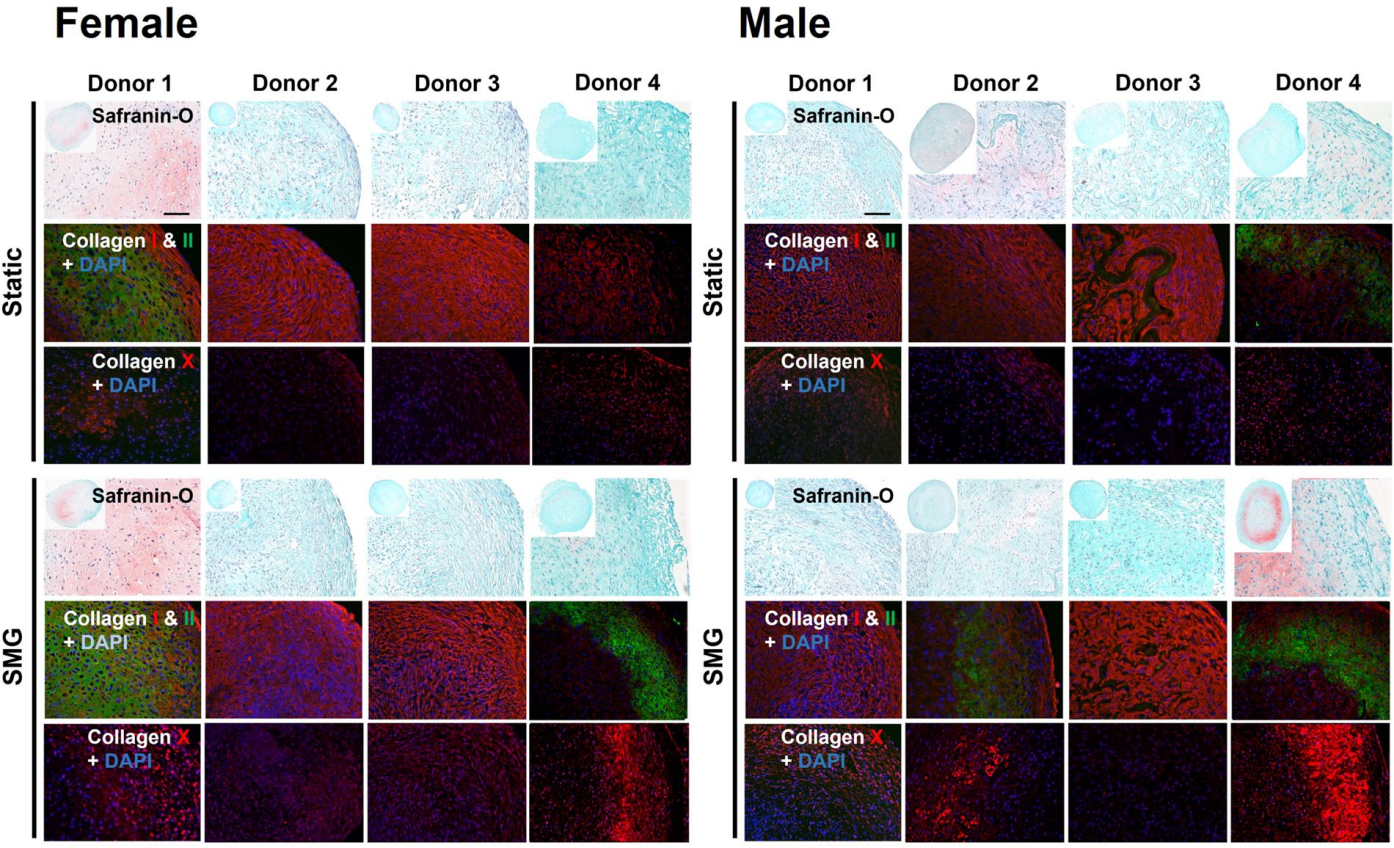
Impact of SMG in the chondrogenic and hypertrophic differentiation potential of engineered meniscus tissues. For each treatment group (static or SMG), top panel: safranin O staining, middle panel: collagen types I, II immunofluorescence staining, bottom panel: collagen type X immunofluorescence staining. Scale bar: 100 μm.

The non-identifiable information of the donors of meniscal specimens are listed in Table 2. After the 2-week preculture and 3-week mechanical stimulation periods, engineered meniscus constructs’ chondrogenic and hypertrophic differentiation potential was qualitatively assessed with Safranin O staining and collagen type I, II, and X immunofluorescence staining (Fig. 2). Donors from both biological sexes exhibited cartilage-like phenotype with a wide range of chondrogenic capacity at the static baseline. Tissues that intensively stained for Safranin O and collagen type II showed more hyaline cartilage-like phenotype, while the rest with strong collagen type I staining showed more fibrous cartilage-like phenotype. The expression levels of collagen type X were comparable for all donors under static conditions. SMG upregulated collagen type X expression in nearly all donors, but the highly variable staining between donors makes it difficult to determine sex differences.

**Table 2:** Meniscal specimen non-identifying donor information.

Sex	Donor Number	Age	Population Doubling (PD)	Sample Code
**Female**	Donor 1	33	2.687	ScACS1.1, ScACS1.2
	Donor 2	44	2.38	ScACS3.1, ScACS3.2
	Donor 3	30	2.774	ScACS4.1, ScACS4.2
	Donor 4	24	1.599	ScAAK9, ScAAK10
**Male**	Donor 1	19	3.349	ScACS2.1, ScACS2.2
	Donor 2	45	2.699	ScACS5.1, ScACS5.2
	Donor 3	22	3.247	ScACS6.1, ScACS6.2
	Donor 4	23	2.263	ScAAK7, ScAAK8

### Quality of whole genome sequencing data

The summary information of the generated RNA-sequencing and processing quality data of each sample is listed in Table 3. The RNA Integrity Number (RIN) of all sequenced samples were close to 10 (10 is the highest possible RNA quality), and the average pre-alignment read quality had Phred scores above 30, indicating high-quality reads. The trimming and alignment algorithm used resulted in an average of at least 95% alignment as well as having the read quality Phred scores maintained. The authenticity of the RNA-sequencing data is also validated by calculating the degree of correlation with the RT-qPCR data of selected genes (Supplementary Fig. 1). An R^2^ value of 0.828 was achieved, showing a strong correlation between the two transcription measurement methods.

**Table 3:** Quality data of RNA-sequencing and bioinformatics processing.

*Sample Name*	*Group*	*RNA extract. method*	*RNA conc. (ng/µL)*	*RIN*	*Pre-alignment*			*Post-alignment*			
					*Total reads*	*Avg. read length*	Avg. quality*	*Total reads*	*Aligned (%)*	*Avg. length*	Avg. quality*
ScAAK7	Static	RNeasy Minikits	1.2	9.1	25994148	42.55	33.60	25851954	95.38	42.45	33.83
ScAAK8	SMG		1.2	9.1	22585197	42.54	33.65	22464022	95.80	42.44	33.87
ScAAK9	Static		2.2	8.6	23676028	42.54	33.65	23550183	96.06	42.44	33.87
ScAAK10	SMG		2.1	8.7	20942143	42.54	33.52	20829886	95.89	42.43	33.74
ScACS1.1	Static	PuroSPIN Total DNA Purification KIT	44	10	21673085	42.50	34.08	21649968	96.20	42.40	34.27
ScACS1.2	SMG		83	9.3	26393415	42.52	34.08	26363008	95.26	42.41	34.32
ScACS2.1	Static		93	9.6	24756374	42.50	34.03	24728910	96.41	42.39	34.23
ScACS2.2	SMG		68	9.5	24302152	42.49	34.06	24276178	96.47	42.38	34.27
ScACS3.1	Static		61	10	20187929	42.48	33.94	20166326	96.42	42.36	34.13
ScACS3.2	SMG		33	9.3	24542608	42.50	34.12	24520924	96.89	42.40	34.25
ScACS4.1	Static		108	10	24586213	42.50	34.10	24563673	96.93	42.39	34.24
ScACS4.2	SMG		156	10	23942119	42.50	34.06	23920514	96.99	42.39	34.21
ScACS5.1	Static		110	9.8	24244159	42.50	34.10	24222229	96.93	42.39	34.24
ScACS5.2	SMG		181	9.9	25369559	42.50	34.06	25347026	96.84	42.39	34.22
ScACS6.1	Static		126	9.7	23496618	42.50	34.12	23473652	96.64	42.40	34.26
ScACS6.2	SMG		175	10	23589464	42.50	34.06	23567332	96.80	42.39	34.21

*Phred Quality Score (−10log_10_Prob) 30: Base call accuracy 99.9%, 40: Base call accuracy 99.99%.

### Donor stratification based on expression level of *COL10A1*

**Fig. 3:**
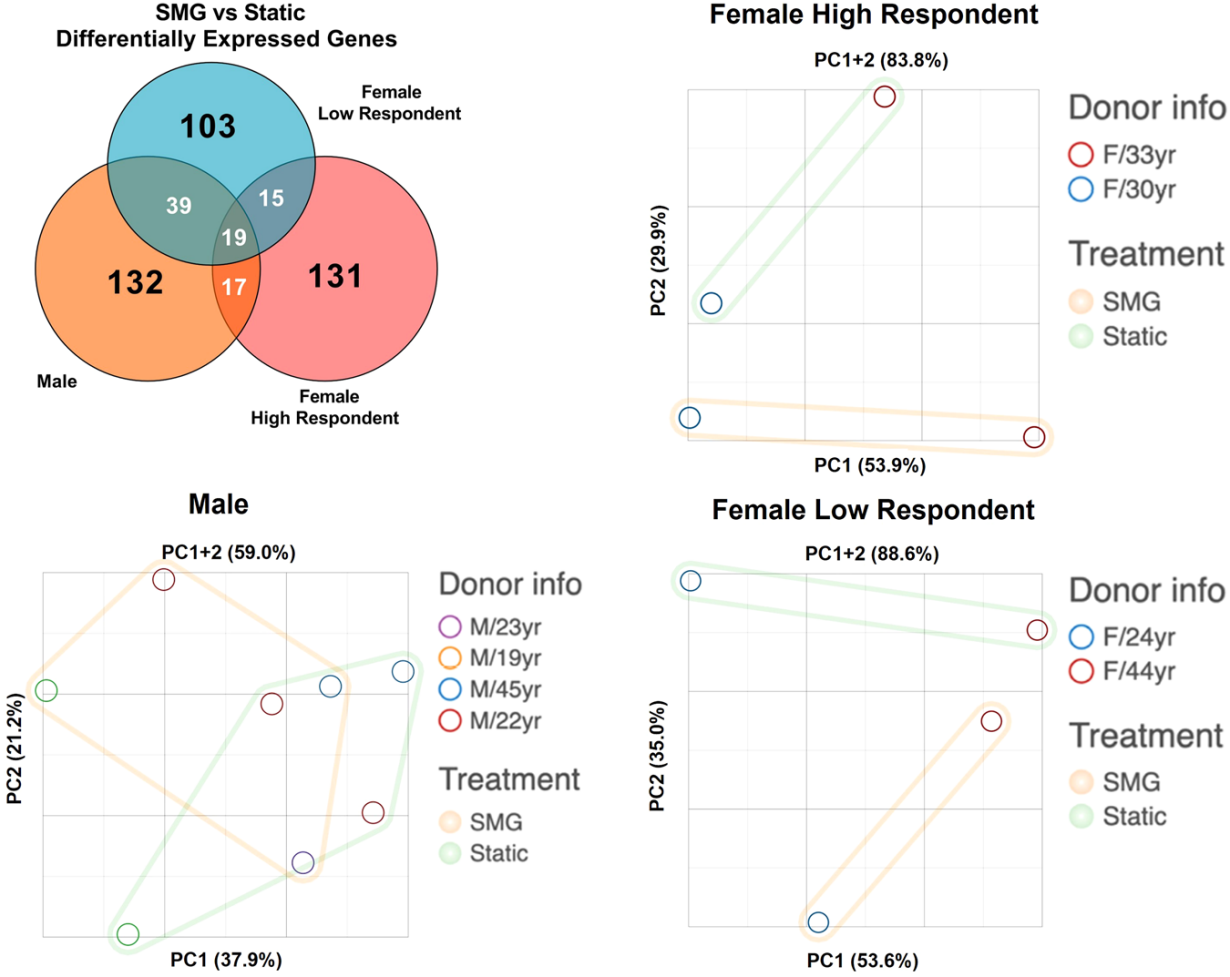
Donor stratification and distinct effect of SMG on sub-groups. DEGs of each group were overlaid and principal component analysis (PCA) was conducted for individual sub-groups.

To account for the effect of the donor variability observed in the previous analyses, a stratification strategy was introduced to separate donors into sub-groups based on the OA-inducing propensity of SMG. Female donors were stratified into high respondents with a higher fold change of *COL10A1* (female donor # 2: 6.84-fold and female donor # 4: 6.78-fold) and low respondents with a lower fold change of *COL10A1* (female donor # 1: 1.56-fold and female donor # 3: 2.17-fold). Male donors all had similar expression fold change of *COL10A1* and remained in one group. The stratification strategy was verified by the Venn diagram showing the overlap of DEGs between the SMG and static in each subgroup and the PCA plot (Fig. 3). All DEGs that appeared in the Venn diagram are listed in Supplement Tables 1–7. The majority of DEGs were unique to each identified group and the samples were well separated by treatment type on the first two principal component (PC) for both female high and female low respondent groups. Further, Table 4 shows a panel of selected OA-related genes that are only significantly modulated in SMG compared to static control for the Female High Respondents cohort. These genes were not significant in the Female Low Respondents or the Male cohorts.

**Table 4:** Select panel of OA-related genes from RNA-sequencing.

Gene	Fold Change for SMG vs Static		
	Female High Respondent	Female Low Respondent	Male
*BMP8A*	8.51**	−1.23 ns	−1.59 ns
*CD36*	11.8*	1.93 ns	1.10 ns
*COL10A1*	6.81***	1.57 ns	1.17 ns
*COL9A3*	5.21*	2.77 ns	−2.32 ns
*FGF1*	3.11*	−2.99 ns	−5.48 ns
*IBSP*	46.1*	1.19 ns	1.21 ns
*IHH*	4.38*	−1.61 ns	−1.99 ns
*MMP10*	43.8**	−3.42 ns	−5.29 ns
*PHOSPHO1*	2.42*	1.34 ns	−1.45 ns
*S100A1*	3.42*	2.01 ns	−2.34 ns
*SPP1*	46.8***	10.9 ns	4.2 ns

*p < 0.05; **p < 0.01; ***p < 0.001; ns = not significant.

## Supplementary information

Supplementary Material

## Data Availability

The bioinformatics software used for this study is Partek® Flow® software (Version 10.0.21.0302, Copyright © 2021, Partek Inc., St. Louis, MO, USA) and the link of online REVIGO tool used is: http://revigo.irb.hr/.
