# Author Correction: Mechanical Unloading of Engineered Human Meniscus Models Under Simulated Microgravity: A Transcriptomic Study

**DOI:** 10.1038/s41597-024-03956-z

**Published:** 2024-10-10

**Authors:** Zhiyao Ma, David Xinzheyang Li, Ryan K. W. Chee, Melanie Kunze, Aillette Mulet-Sierra, Mark Sommerfeldt, Lindsey Westover, Daniel Graf, Adetola B. Adesida

**Affiliations:** 1 Department of Surgery, Division of Orthopaedic Surgery, Faculty of Medicine & Dentistry, University of Alberta, Edmonton, AB T6G 2E1 Canada; 2 Department of Civil & Environmental Engineering, University of Alberta, Edmonton, AB Canada; 3 Department of Mechanical Engineering, University of Alberta, Edmonton, AB Canada; 4 School of Dentistry, University of Alberta, Edmonton, AB Canada

**Keywords:** Mechanisms of disease, Data acquisition

Correction to: *Scientific Data* 10.1038/s41597-022-01837-x, published online 30 November 2022

In the version of the article initially published, in Table 2, the age of the female donor 4 was originally listed as “31” and has now been amended to “24”, and the age of the male donor 4 was originally “59” and has now been amended to “23”. These corrections have been made to the HTML and PDF versions of the article.

