# Supplementary material for: Mechanical Unloading of Engineered Human Meniscus Models Under Simulated Microgravity: A Transcriptomic Study
Source: Sci Data. 2022 Nov 30;9:736. doi: 10.1038/s41597-022-01837-x (PMC9712603; doi:10.1038/s41597-022-01837-x)
Supplement: Supplementary file 1 — Supplementary Material [file 41597_2022_1837_MOESM1_ESM.pdf]

Content:

Supplement Figure 1

Supplement Table 1-7

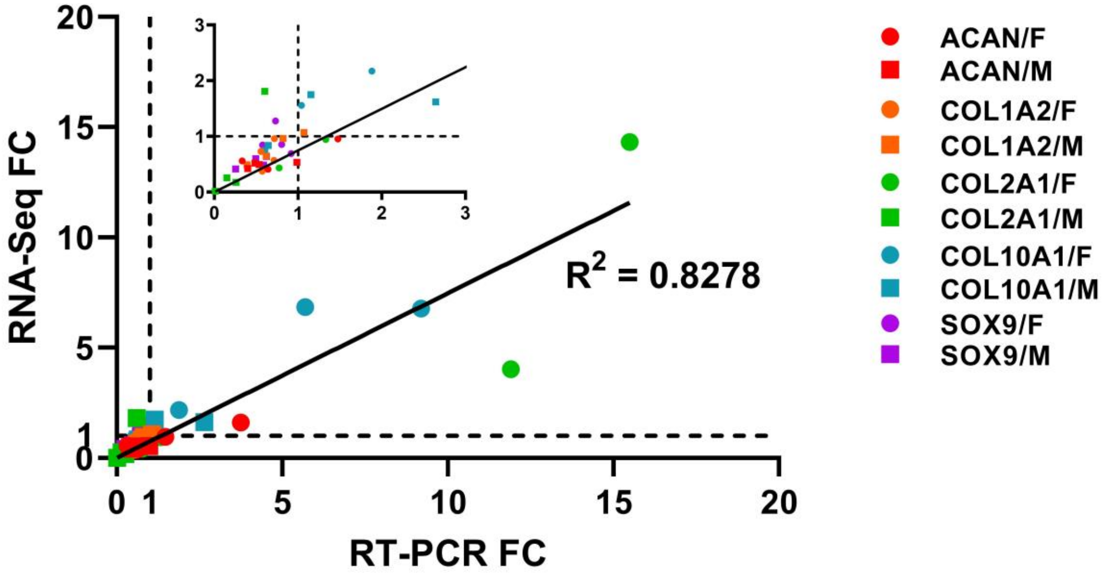

Supplemental Figure 1. Correlation validation of RT-qPCR and RNA-Seq data. The value was reported as fold change (SMG to Static). Each data point corresponded to a single donor.

Supplementary Table 1. RNA sequencing genes from Venn diagram in Figure 3. All Female High Respondent genes (182 genes).

Gene symbol	**P-value (SMG vs. Static)**	**Fold change (SMG vs. Static)**
ADAMTS3	2.42E-02	-3.423
ADAMTSL2	5.46E-03	-14.869
ADIRF	1.53E-02	-3.872
AKAP12	4.20E-02	2.433
AKR1C1	3.69E-02	2.109
AKR1C3	6.44E-03	2.419
ALDOC	2.97E-03	-2.759
ALPK3	3.52E-02	-2.842
ANK2	5.48E-03	2.328
APOL6	4.64E-02	2.529
AQP1	3.72E-02	-2.822
ATF3	1.92E-02	3.623
BCL2	2.90E-02	2.019
BEST1	4.99E-03	2.496
BIRC3	2.25E-02	7.336
BMP8A	3.51E-03	8.510
BNIP3	1.99E-02	-2.486
C15orf48	1.71E-02	61.319
C8orf44-SGK3	2.47E-02	2.278
CA9	2.20E-03	-5.625
CACNA1D	3.91E-02	-2.556
CACNA1H	3.05E-03	8.510
CACNA1I	1.51E-02	-6.057
CADM1	8.69E-03	-2.184
CADPS	3.74E-02	-3.099
CADPS2	2.35E-02	2.706
CD36	1.07E-02	11.767
CELSR3	3.85E-02	-3.168
CHRFAM7A	1.16E-02	-2.477
CHST6	3.30E-02	-2.097
CILP2	4.28E-02	-2.523
CKB	2.64E-02	-2.252
CNN1	1.26E-02	-2.620
CNTN1	2.59E-02	-2.803
COL10A1	6.38E-04	6.809
COL2A1	3.33E-03	9.440
COL9A3	2.97E-02	5.210
CPT1A	8.94E-03	3.418
CRABP2	1.31E-02	-3.903
CRTAC1	7.94E-03	5.569
CRYGS	4.15E-02	2.371
CSRP2	1.21E-02	-2.825
CTSH	1.97E-03	3.566
CTSS	3.54E-02	2.473
CXCL13	3.75E-02	3.633
CYP26A1	4.82E-03	-11.812
CYP4V2	1.91E-02	2.225
DDIT4L	3.55E-02	-4.238
DIRAS2	4.60E-02	14.613
DLX5	2.98E-02	-2.191
DRD4	1.02E-02	-3.059
DSG3	4.56E-03	3.857
DTX3L	1.45E-02	2.183
DUSP10	3.40E-02	2.171
EDNRA	1.03E-02	2.218
EGR2	1.93E-02	2.966
EPGN	2.40E-03	-4.432
ERAP2	1.85E-02	2.118
EVI2A	8.78E-03	2.884

EVI2B	4.11E-02	2.668
FAM156A	2.74E-02	-2.504
FAM81A	4.55E-02	4.847
FBN2	3.83E-02	-3.511
FER1L4	2.45E-03	-4.478
FFAR4	1.33E-02	4.797
FGF1	2.03E-02	3.112
FILIP1	4.21E-02	2.661
FOXA2	2.17E-02	30.869
FRMD7	3.25E-02	2.217
FRRS1	9.03E-03	2.426
FRY	3.73E-02	2.196
FTH1	3.41E-02	2.248
FTH1P3	9.69E-03	2.215
GOLGA8T	2.08E-02	-2.930
GPNMB	5.47E-03	2.393
GPR171	3.31E-03	11.314
H19	3.47E-03	-4.481
HCFC1R1	8.78E-03	-2.013
HHIPL2	3.96E-02	2.804
HMOX1	3.49E-02	2.426
HOTS	4.25E-03	-4.015
HP09053	4.61E-02	-2.585
HRCT1	4.85E-03	-4.278
HSPB7	8.37E-03	-2.948
IBSP	3.23E-02	46.069
IGF2	3.22E-02	-2.918
IGFL3	2.43E-02	-5.124
IHH	1.78E-02	4.382
ISM1	2.31E-02	-2.629
JAK2	3.12E-02	2.116
JMJD7-PLA2G4B	2.24E-02	-5.267
JPH1	3.56E-02	5.735
KITLG	4.18E-03	3.532
KLRD1	7.17E-03	14.101
KRT75	8.11E-04	7.575
LOC100130111	2.14E-02	-2.637
LOC112694756	7.41E-03	-2.338
LOXL2	1.22E-02	-2.435
MAP3K5	3.15E-02	2.371
MBNL3	1.54E-02	2.342
MFAP5	3.16E-02	-2.743
MIF	4.77E-03	-2.486
MIF-AS1	2.85E-03	-2.270
MIR210HG	5.62E-03	-2.925
MLPH	1.60E-02	2.636
MME	2.07E-02	2.037
MMP10	4.41E-03	43.752
MYCL	2.09E-02	-3.378
NCAM1	4.57E-02	-2.297
NCR3LG1	4.71E-02	2.035
NDUFA4L2	3.34E-04	-4.887
NEDD9	4.71E-02	-2.092
NOS2	3.24E-02	3.944
NPTX2	7.26E-03	5.090
NQO1	3.87E-02	2.266
NRG1	4.23E-04	-7.824
NXPH3	1.01E-02	-2.064
ODAPH	1.01E-03	-21.045
OPCML	3.17E-02	2.675
OTULINL	3.59E-02	-2.048
PALM	5.08E-03	-2.070
PAPPA2	3.78E-02	3.447
PBK	4.87E-02	2.064

PDE7B	4.57E-02	2.188
PGK1	2.18E-02	-2.214
PHLDA1	1.66E-02	2.941
PHOSPHO1	4.12E-02	2.421
PKNOX2	4.80E-02	-2.714
PLIN2	4.96E-03	2.113
PMAIP1	4.07E-02	2.707
PODN	3.39E-02	2.032
PPEF1	4.81E-02	3.564
PRSS53	4.26E-02	-2.312
RAB11FIP4	2.90E-03	4.008
RAC3	2.82E-02	-2.219
RAD54B	4.10E-02	2.552
RASGEF1A	1.43E-02	-3.028
RASSF7	1.97E-02	-2.456
REEP2	4.43E-02	-2.067
RIPOR2	3.55E-02	-2.338
RSPO2	8.14E-03	-3.160
RYR2	4.66E-02	-4.324
S100A1	3.74E-02	3.435
S100A13	3.71E-02	2.441
S1PR1	3.36E-02	2.409
S1PR3	2.41E-02	2.353
SCARF2	6.04E-03	-2.620
SCD5	4.28E-02	2.024
SCIN	2.75E-02	4.910
SCN2A	2.90E-02	4.290
SCN3B	2.75E-02	5.335
SCT	1.16E-02	-2.844
SEMA6A	1.98E-02	-2.165
SFRP2	1.46E-02	-4.896
SHANK2	4.42E-03	-4.253
SLC13A5	1.53E-02	15.941
SLC20A1	7.45E-03	2.038
SLC44A2	2.71E-02	2.044
SMG1P1	2.36E-02	2.295
SNX15	2.00E-02	4.463
SOD2	1.53E-02	2.889
SPINT2	4.82E-02	-2.124
SPP1	2.06E-05	46.806
STAC	1.21E-02	-3.048
STON2	2.81E-03	3.681
SUSD4	3.61E-02	8.985
TFR2	1.90E-02	-5.959
TFRC	1.35E-02	2.088
TGM2	2.67E-02	8.481
THBS4	2.23E-02	-3.333
TMEM150C	7.29E-03	2.513
TMEM200A	1.30E-02	2.280
TMEM200C	2.16E-02	9.530
TNFAIP6	3.41E-02	2.747
TNNT3	4.96E-02	-2.087
TPI1	7.75E-03	-2.139
TRIL	2.35E-02	-2.329
TRIM47	4.68E-02	2.575
TRIM9	4.38E-02	2.142
UBE2QL1	2.10E-02	-2.724
VSNL1	2.65E-03	-3.900
ZNF385B	1.98E-02	3.737

Supplementary Table 2. RNA sequencing genes from Venn diagram in Figure 3. All Female Low Respondent genes (176 genes).

Gene symbol	**P-value (SMG vs. Static)**	**Fold change (SMG vs. Static)**
ABCD2	3.65E-02	3.287
ABCG1	1.38E-02	-5.955
ACAT2	1.74E-02	3.159
ADAMTS3	1.64E-02	-2.619
ADAMTS9-AS2	3.67E-02	-3.538
ADSS1	3.28E-02	-4.551
AJM1	1.88E-02	-3.518
AKR1C1	2.40E-02	2.425
ALPK3	1.07E-02	-3.621
APLN	1.13E-02	-6.595
APOE	5.80E-03	-3.696
APOL6	4.98E-03	3.326
ARHGEF19	2.47E-02	-2.318
ATF3	4.85E-02	2.654
B4GALNT4	1.48E-02	-2.190
BCKDHA	3.62E-02	-2.108
BEST1	7.22E-03	2.651
BMP2	1.02E-02	2.322
BRSK1	2.29E-02	-2.875
CACNA1I	7.16E-03	-19.735
CAPG	2.90E-02	3.368
CASP1	2.82E-02	8.901
CBR3	1.72E-02	2.905
CCN3	1.40E-02	-3.517
CDK15	3.72E-02	-2.582
CDKN2A	3.41E-02	2.013
CHST6	1.43E-02	-2.038
CILP2	2.86E-02	-2.803
CLEC18A	4.65E-02	-2.210
CMKLR1	1.11E-02	2.467
COL13A1	2.74E-02	-2.891
COMMD3-BMI1	3.78E-02	2.347
CPNE7	1.78E-02	10.113
CPZ	1.80E-02	-2.137
CRABP2	5.56E-03	-3.680
CRIP1	9.30E-04	18.303
CSRP2	9.54E-03	-2.089
CST7	4.09E-02	2.966
CTAGE4	4.34E-02	-11.451
CTSH	2.73E-02	4.797
CX3CL1	4.32E-02	-2.550
CYP26A1	3.51E-02	-4.452
CYP4V2	3.49E-02	2.121
CYP51A1	3.69E-02	2.414
DDIT4L	2.07E-02	-3.080
DEPP1	9.36E-03	-7.242
DHRS4L2	4.52E-02	3.319
DLG4	3.10E-02	-2.262
DLX5	3.22E-02	-2.344
DLX6	3.47E-02	-2.065
DMD	4.32E-02	2.398
EGLN3	2.05E-03	-6.740
EGR2	1.96E-02	2.971
ENO2	1.66E-02	-3.853
EPGN	2.05E-02	-5.078
EPOR	4.90E-02	-2.288
ERMN	3.97E-02	5.251
ETV5	1.55E-02	2.200
EXOSC6	2.83E-02	2.392
F2R	3.16E-02	2.518

F3	6.61E-03	2.618
FABP3	2.65E-02	2.365
FABP4	3.04E-03	19.322
FAM155A	6.14E-03	9.242
FDFT1	4.33E-02	2.020
FER1L4	1.24E-02	-11.811
FGF11	2.21E-02	-2.904
FGFR2	9.77E-03	-2.406
FRMPD3	2.70E-02	-3.541
FTH1	2.86E-02	3.707
FTH1P3	5.26E-03	3.159
GABBR1	4.62E-02	-2.011
GCK	3.03E-02	-4.284
GCLM	3.66E-02	2.435
GFRA2	1.33E-02	-2.154
GJA5	2.29E-02	-2.022
GOLGA2P10	3.28E-02	-2.100
GOLGA8A	5.22E-03	-2.353
GOLGA8O	1.63E-02	-2.866
GPR1	3.53E-02	2.217
GSDMB	1.51E-02	-4.471
GUSBP4	4.67E-02	-2.830
HACD1	4.81E-02	2.108
HMGA2	3.65E-02	2.445
HMGCS1	2.53E-02	2.097
HMOX1	4.87E-02	3.548
HSD11B2	3.44E-04	-22.627
HSD17B7	2.37E-02	2.690
HSF4	2.53E-02	-2.192
HSPA2	1.83E-02	-2.135
ICAM5	2.18E-02	-12.788
IDH1	4.81E-02	2.068
IFI6	4.10E-02	2.568
IFIT1	8.97E-03	2.814
IFIT2	7.06E-03	4.116
IFIT3	1.41E-02	4.041
IGFN1	2.03E-02	-9.119
INHBB	2.61E-02	-2.884
JPH2	3.54E-02	-4.002
KCNAB1	4.34E-02	-2.388
KCTD11	4.67E-02	-2.176
KHDRBS3	3.34E-02	2.774
KRT14	5.48E-03	-3.383
LDLR	3.71E-03	2.732
LFNG	2.80E-02	2.446
LINC00641	1.54E-02	-3.019
LOC112694756	5.91E-03	-2.656
LOC143666	1.83E-02	-4.990
LOC154761	7.76E-03	-3.515
LRP8	2.98E-02	2.046
MAPK13	3.77E-02	-3.094
MLPH	2.56E-02	3.452
MMP3	1.98E-02	-2.593
MOB3B	3.14E-02	-2.180
MSMO1	2.34E-02	2.598
MTRNR2L2	1.77E-02	2.120
MYLIP	5.90E-03	-2.501
NAGPA	2.43E-02	2.782
NEDD9	2.02E-02	-2.028
NEFL	4.56E-02	2.518
NEXN	4.62E-02	2.757
NKD1	9.35E-04	-3.693
NMRAL2P	3.28E-02	9.065
NPHP3-ACAD11	4.12E-03	-13.402

NQO1	5.00E-03	3.670
NUDT4B	3.41E-02	2.230
OBSCN	2.64E-02	-3.260
PCDH1	1.90E-02	3.306
PHLDA2	1.51E-02	4.256
PIM1	3.25E-03	-2.762
PLCL1	9.22E-03	4.831
PMAIP1	1.49E-02	2.690
PPFIA4	3.71E-02	-3.826
PPT2-EGFL8	2.74E-02	-2.695
PRKAA2	4.59E-02	-4.994
PRSS53	1.08E-02	-3.666
PRXL2A	2.43E-02	2.110
PTGS1	3.53E-02	-3.285
RAP1GAP2	2.99E-02	-2.743
RAPGEF4	3.38E-02	-2.279
RASD2	4.84E-02	-16.994
RASGEF1A	1.97E-02	-3.665
RDH10	3.71E-02	2.214
RNA45SN2	3.45E-02	3.073
RSPO2	9.70E-03	-3.462
SAMHD1	4.54E-02	3.427
SDHAP1	2.77E-02	-2.230
SELENOP	5.65E-03	2.824
SERPINI1	4.39E-02	3.070
SH3D21	1.67E-02	-5.221
SLC20A1	2.73E-03	2.886
SLC22A17	3.06E-02	-2.165
SLC24A2	2.21E-02	2.515
SLC2A12	3.45E-03	-3.450
SLC2A3	4.99E-02	-2.240
SLC2A5	2.34E-02	-9.276
SLC31A2	3.04E-02	2.397
SMAGP	4.89E-02	2.249
SMIM4	4.78E-02	2.261
SP110	3.71E-02	2.014
SQSTM1	4.94E-02	2.052
STAC	2.87E-02	-2.684
STARD4-AS1	1.78E-02	-2.821
SYNPO	9.86E-04	-3.750
TALAM1	3.82E-03	-4.053
TFRC	4.99E-03	2.327
THBS3	1.49E-02	-2.050
TMEM154	3.54E-02	2.044
TNFRSF9	3.32E-02	-2.470
TPPP3	1.23E-02	-3.838
TRIM47	2.29E-02	3.006
VEGFA	3.13E-02	-9.211
WSB1	2.66E-02	-2.130
WWC1	3.60E-02	-2.979
ZDHHC23	2.22E-02	3.353
ZNF395	4.69E-02	-2.798

Supplementary Table 3. RNA sequencing genes from Venn diagram in Figure 3. All Male genes (207 genes).

Gene symbol	**P-value (SMG vs. Static)**	**Fold change (SMG vs. Static)**
ACAT2	1.21E-02	2.436
ACSM3	4.00E-02	-2.172
ADAMTS7	4.12E-03	2.517
ADAMTS9-AS2	3.28E-03	-4.522
ADGRL4	4.61E-02	2.176
ADM	8.01E-03	-2.538
ADRA2A	3.17E-03	2.363
ADSS1	7.16E-04	-4.618
AGAP11	1.12E-02	-2.665
AK4	1.56E-02	-2.866
ALDOC	2.88E-02	-2.583
ALPK3	1.83E-02	-2.318
ANGPTL6	4.60E-02	-7.153
ANKRD29	4.81E-02	2.330
APLN	1.64E-02	-4.454
ARRDC4	2.69E-02	-2.209
BCL6B	1.14E-02	-3.886
BEST1	9.19E-03	2.120
BEST4	4.95E-02	-4.436
BNIP3	3.54E-02	-3.226
C3	1.96E-02	-4.004
C3AR1	5.82E-03	2.951
CA9	1.80E-02	-14.504
CACNA1I	4.38E-02	-13.636
CAPG	1.04E-03	3.120
CCDC80	1.96E-02	-2.031
CCN1	8.33E-03	-2.043
CHI3L2	3.88E-02	-2.532
CHRNA9	4.92E-02	4.915
CILP2	3.89E-02	-3.176
CLEC3B	2.34E-02	-4.533
CLMAT3	1.90E-02	-2.352
CLU	6.69E-03	-2.423
COBL	1.45E-02	8.192
CPNE7	3.16E-02	9.510
CPT1A	1.22E-02	2.356
CRABP2	3.84E-02	-2.766
CRIP1	1.94E-03	11.749
CSF1	8.94E-03	2.250
CYP26B1	1.91E-02	-3.139
DDIT4L	3.52E-02	-4.577
DEPP1	1.78E-03	-4.661
DHCR7	2.38E-02	2.003
DHRS13	3.06E-02	-2.392
DHRS3	3.98E-02	-5.452
DRD4	4.77E-03	-2.415
DUSP27	2.44E-02	-3.875
DUSP6	1.75E-02	2.547
EGLN3	3.97E-03	-10.470
EGR2	1.22E-02	2.120
ELOVL3	2.33E-02	-2.451
EPB41L4A	4.00E-02	-2.872
EPB41L4A-AS1	1.48E-02	-2.159
EPGN	4.91E-02	-4.134
ETV1	4.59E-02	2.006
EYA4	5.38E-03	2.066
FAM155A	4.62E-03	7.286
FBLN7	4.86E-03	-2.874
FNDC10	5.11E-03	2.579

FRMPD3	2.74E-02	-2.908
FTH1	2.07E-02	2.289
FTH1P3	1.75E-02	2.255
GCLM	1.25E-02	2.281
GDF15	4.53E-02	2.256
GDF5	1.24E-02	-2.674
GET1-SH3BGR	3.60E-02	-2.070
GMPR	4.34E-02	-2.363
GPR1	7.46E-03	2.659
GPR146	4.90E-02	-2.594
GPR183	3.16E-02	-3.270
GPR68	3.51E-02	2.282
H19	4.60E-02	-10.826
HILPDA	3.34E-02	-5.886
HK2	2.66E-02	-3.211
HMGA2	4.18E-02	2.043
HRCT1	1.38E-02	-4.145
HSD11B2	2.39E-03	-14.326
HSD17B7	1.89E-02	2.246
HSPA2	7.51E-03	-2.082
HTR7	2.72E-03	3.722
IGF2	1.39E-02	-7.089
IGFL3	3.66E-02	-3.067
IGFN1	3.27E-02	-9.087
INHBB	4.71E-02	-2.087
IRX3	3.17E-02	2.599
ISLR2	4.79E-02	2.875
ITGA3	1.77E-03	3.091
ITPR3	7.56E-05	2.737
ITPRIP	3.38E-02	2.314
JAM2	4.54E-02	-2.390
JPH2	1.19E-02	-4.429
JPH3	1.09E-03	2.241
KCNAB1	2.17E-03	-2.305
KCNC4	2.85E-03	2.083
KCNH1	3.31E-02	2.185
KLHL4	1.73E-02	2.139
KLRD1	6.04E-03	7.030
LDLR	2.20E-03	2.290
LEFTY2	1.69E-02	-3.845
LINC00641	1.80E-02	-2.168
LINC00856	1.39E-02	2.982
LINC02067	1.23E-02	-2.262
LOC100310756	2.61E-02	-2.016
LOC100507412	2.21E-02	2.026
LOC112694756	2.60E-02	-2.002
LOC154761	7.23E-03	-2.343
MAPK13	2.17E-03	-2.619
MCOLN3	1.68E-03	3.561
MFAP5	1.69E-02	-2.076
MICB	1.10E-02	2.890
MIR210HG	3.86E-02	-3.477
MLPH	6.53E-06	4.039
MMP15	3.47E-03	2.912
MMP23A	1.50E-02	-5.482
MMP23B	6.83E-04	-5.214
MMP3	4.87E-03	-7.375
MSTO2P	4.67E-02	2.220
MYLIP	8.02E-03	-2.506
NCR3LG1	1.07E-03	2.016
NDUFA4L2	2.73E-02	-4.242
NEXMIF	4.47E-02	-2.815
NFASC	1.18E-03	2.071
NOL3	3.62E-02	-2.693

NPL	1.77E-02	2.364
NPR3	3.28E-02	-3.190
NPTX1	2.93E-02	-16.142
NQO1	6.01E-03	2.576
NR2F1	1.00E-02	2.205
NRN1	2.41E-02	-2.417
NTM	2.71E-02	2.421
NTNG2	1.29E-02	2.026
NXPH4	4.93E-02	-3.021
NYAP1	2.04E-02	-3.472
OLFML2A	1.33E-02	4.798
PAK3	2.72E-02	2.321
PCDH1	5.22E-03	3.298
PDK1	1.15E-02	-3.640
PER2	1.71E-02	-2.280
PFKFB3	3.03E-03	-2.294
PGAM2	1.60E-02	-2.407
PHLDA2	1.45E-02	2.746
PIANP	1.24E-02	-3.019
PIK3AP1	5.80E-03	-2.177
PLAT	7.19E-03	2.326
PMAIP1	3.81E-02	2.281
PPFIA4	3.67E-02	-2.824
PRELID2	8.51E-03	-2.986
PRELP	3.63E-02	-2.487
PTCHD4	2.18E-03	3.551
PTGS1	5.26E-03	-3.616
RAB20	3.47E-02	-2.560
RASD2	3.15E-02	-3.822
RASEF	8.76E-03	4.666
RASL10B	1.06E-02	-2.643
RASSF4	1.59E-02	-2.971
RGL3	6.98E-03	-2.193
RHOH	7.29E-03	2.658
RIPOR2	1.29E-02	-2.558
RNF125	3.46E-02	2.191
RSPO2	6.30E-04	-4.260
SBSPON	3.88E-02	-10.479
SCN2A	3.99E-02	3.384
SCT	2.10E-02	-2.566
SDSL	2.51E-02	2.052
SEMA3B	1.63E-02	-3.523
SERINC2	5.78E-03	2.159
SERPINA5	6.66E-04	-2.491
SGCA	1.68E-02	-3.549
SLC20A1	5.27E-03	2.493
SLC22A3	1.76E-02	4.193
SLC24A2	9.85E-03	9.551
SLC2A1	1.19E-02	-2.313
SLC2A5	2.09E-02	-8.837
SLC2A6	1.23E-02	2.259
SLC31A2	1.73E-04	2.106
SLC4A11	6.78E-03	4.120
SLC5A12	2.38E-02	-5.714
SLC8A1	1.23E-02	-2.898
SMOC2	7.96E-03	-2.750
SNORD141A	3.48E-02	-2.033
SNORD141B	3.48E-02	-2.033
SPAG4	3.19E-02	-3.540
SPHK1	5.15E-03	2.962
SRPK3	2.49E-02	-2.231
ST6GALNAC3	4.34E-02	-3.460
STAC	1.17E-02	-3.198
SYNPO	1.56E-02	-3.781

SYT12	2.58E-02	3.325
TCAF2	1.98E-02	-2.224
TESK2	8.68E-03	-2.020
TET1	9.06E-03	-2.491
TFRC	2.02E-02	2.052
THSD1	1.76E-03	3.145
TRIM29	9.25E-03	-2.677
TRIM47	1.17E-02	2.126
TUBB2B	2.01E-02	-4.491
TXNIP	1.78E-02	-2.316
VAV3	4.52E-02	2.006
VSIR	8.71E-03	-2.735
VWA1	1.53E-03	-2.893
WHRN	3.36E-02	2.246
WNT9A	6.75E-03	3.317
WWC1	3.86E-02	-3.058
ZDHHC2	1.03E-02	3.073
ZDHHC23	2.97E-02	2.148
ZNF185	3.90E-02	-2.972
ZNF395	7.82E-04	-3.005

Supplementary Table 4. RNA sequencing genes from Venn diagram in Figure 3. Female High Respondents AND Female Low Respondents genes (34 genes).

Gene symbol
ADAMTS3
AKR1C1
ALPK3
APOL6
ATF3
BEST1
CACNA1I
CHST6
CILP2
CRABP2
CSRP2
CTSH
CYP26A1
CYP4V2
DDIT4L
DLX5
EGR2
EPGN
FER1L4
FTH1
FTH1P3
HMOX1
LOC112694756
MLPH
NEDD9
NQO1
PMAIP1
PRSS53
RASGEF1A
RSPO2
SLC20A1
STAC
TFRC
TRIM47

Supplementary Table 5. RNA sequencing genes from Venn diagram in Figure 3. Female High Respondents AND Male genes (36 genes).

Gene symbol
ALDOC
ALPK3
BEST1
BNIP3
CA9
CACNA1I
CILP2
CPT1A
CRABP2
DDIT4L
DRD4
EGR2
EPGN
FTH1
FTH1P3
H19
HRCT1
IGF2
IGFL3
KLRD1
LOC112694756
MFAP5
MIR210HG
MLPH
NCR3LG1
NDUFA4L2
NQO1
PMAIP1
RIPOR2
RSPO2
SCN2A
SCT
SLC20A1
STAC
TFRC
TRIM47

Supplementary Table 6. RNA sequencing genes from Venn diagram in Figure 3. Female Low Respondents AND Male genes (58 genes).

Gene symbol
ACAT2
ADAMTS9-AS2
ADSS1
ALPK3
APLN
BEST1
CACNA1I
CAPG
CILP2
CPNE7
CRABP2
CRIP1
DDIT4L
DEPP1
EGLN3
EGR2
EPGN
FAM155A
FRMPD3
FTH1
FTH1P3
GCLM
GPR1
HMGA2
HSD11B2
HSD17B7
HSPA2
IGFN1
INHBB
JPH2
KCNAB1
LDLR
LINC00641
LOC112694756
LOC154761
MAPK13
MLPH
MMP3
MYLIP
NQO1
PCDH1
PHLDA2
PMAIP1
PPFIA4
PTGS1
RASD2
RSPO2
SLC20A1
SLC24A2
SLC2A5
SLC31A2
STAC
SYNPO
TFRC
TRIM47
WWC1
ZDHHC23
ZNF395

Supplementary Table 7. RNA sequencing genes from Venn diagram in Figure 3. Female High Respondents AND Female Low Respondents AND Male genes (19 genes).

Gene symbol
ALPK3
BEST1
CACNA1I
CILP2
CRABP2
DDIT4L
EGR2
EPGN
FTH1
FTH1P3
LOC112694756
MLPH
NQO1
PMAIP1
RSPO2
SLC20A1
STAC
TFRC
TRIM47
